# KNG-1, HSPG and Robo4 reflect hypertension-driven subclinical renal alterations and provide incremental diagnostic value in type 2 diabetic nephropathy

**DOI:** 10.1038/s41598-026-57087-z

**Published:** 2026-06-17

**Authors:** Heba Ibrahim Hamed, Ashraf Ismail Amin, Heba Ramadan Ahmed, Ahmed M. A. Akabawy

**Affiliations:** 1 Biochemistry Department, Research and training unit, National Institute of Diabetes and Endocrinology, General Organization for Teaching Hospitals and Institutes, Cairo, Egypt; 2 Clinical Pathology Department, National Institute of Diabetes and Endocrinology, General Organization for Teaching Hospitals and Institutes, Cairo, Egypt; 3 Internal Medicine Department, National Institute of Diabetes and Endocrinology, General Organization for Teaching Hospitals and Institutes, Cairo, Egypt; 4 Biochemistry and Molecular Biology Department, Faculty of Pharmacy, Capital University (Formerly Helwan University), Ain Helwan 11795, Helwan, Cairo Egypt

**Keywords:** Diabetic nephropathy, Hypertension, KNG-1, HSPG, Robo4, T2DM, Biomarkers, Diseases, Endocrinology, Nephrology

## Abstract

Diabetic nephropathy (DN), indicated by persistent urinary albumin excretion or albuminuria, is the major cause of end stage renal disease worldwide. Currently albuminuria is considered the best predictor of subsequent development of DN in type 2 diabetic patients, but it is often induced by arterial hypertension or heart failure in older patients as well. Therefore, additional biomarkers are needed to identify diabetic patients at a high risk of developing DN. The aim of the study was to investigate the expression profiles of some serum proteome biomarkers and to demonstrate their association with early renal alterations among type 2 diabetic patients. This study included 300 type 2 diabetic patients with- (200, UACR > 30 mg/gm) and without- (100, UACR < 30 mg/gm) persistent albuminuria in addition to 100 healthy control subjects. Serum expression patterns of KNG-1, HSPG, Robo4 and other biochemical parameters were evaluated in all participants. We also assessed the combined influence of hypertension and albuminuria on these markers, to explore their potential as non-invasive indicators of early renal risk in T2DM. Results suggest that elevated serum KNG-1, HSPG and Robo4 levels in type 2 diabetic patients might reflect a DN progressive pathology due to various mechanisms. The interaction between hypertension and albuminuria significantly alters biomarker levels in a synergistic manner, emphasizing the need for comprehensive management of both conditions in clinical practice. Additionally, findings highlight that serum proteome signature of these biomarkers provide an incremental predictive value when integrated with conventional clinical parameters, as evidenced by the marked improvement in multivariable regression model performance and discriminative accuracy analyses.

## Introduction

 Diabetes mellitus (DM) is a major public health problem worldwide. Diabetes-related mortality and morbidity are associated with its chronic complications, either vascular or non-vascular. DM and its complications affect individuals, families, healthcare systems, and national economies^1^. Type 2 diabetes mellitus (T2DM) is a global healthcare concern. About one‑third of these patients are likely to develop diabetic nephropathy (DN), which is devastating in terms of clinical, economic, and ethical dimensions^2^. DN is a leading cause of morbidity and mortality among patients with T2DM. It is characterized by decreased glomerular filtration rate (GFR) with intraglomerular hypertension and clinically progressive albuminuria, followed by eventual loss of renal function^3^. Changes in GFR or albuminuria are currently considered hallmarks of onset or progression of DN. However, levels of estimated GFR (eGFR) or urinary albumin are still in the normal ranges in some patients with early stages of underlying nephropathy pathological changes, which if not considered, it will progress irreversibly. This suggests that eGFR and/or albuminuria may not be the most suitable predictor for early diagnosis of DN. This has motivated researchers to consider potential novel clinically diagnostic biomarkers^4^. Kininogen is a forerunner of kinins from the kallikrein-kinin system (KKS), which are relevant to cardiovascular and renal function, blood pressure regulation and many physiological and pathological processes^5^. The orthological proteins (kininogen-1 (KNG-1), UniProt ID: P01042 for human) are produced by alternative splicing of the KNG1 gene transcript^6^. Basement membrane specific Heparan sulfate proteoglycan (HSPG) is a sulfated linear polysaccharide containing repeating disaccharides of *N*-acetylatedglucosamine and uronic acid. Heparan Sulfate (HS) chains are synthesized directly onto a core protein forming a HSPG, e.g., collagen type XVIII (COL18), syndecans (SDC1-4), glypicans (1–6), agrin, CD44 and perlecan. The core proteins are essential to HS synthesis because they provide the necessary serine residues required to initiate HS assembly. While HSPG core proteins undergo folding and maturation in the endoplasmic reticulum, HS chains are assembled onto the core proteins in the Golgi Apparatus and subsequently undergo chemical modification. HS in beta cells functions as a non-enzymatic antioxidant influencing postnatal islet maturation and regulating insulin secretion^7^. Roundabout homolog 4 (Robo4) is a member of the Robo family, which is involved in angiogenesis maintenance and vessel integrity. Robo4 participates in physiological angiogenesis and promotes vessel maturation. However, in pathogenic angiogenesis, Robo4 has been found to accelerate the progression of angiogenesis^8^. In this research, we aimed at investigating the expression profiles of some serum proteome biomarkers, KNG1, HSPG and Robo4 among type 2 diabetic patients with and without DN. Additionally, we aimed also at identifying whether these serum biomarkers, alone or in combination, could serve as a simple, non-invasive utility for assessing the risk of renal functional decline within T2DM patients especially in those at higher risk due to coexisting hypertension. Detection of such changes in earlier stages could help delay or prevent the progression of DN and reduce the risk of end-stage renal disease (ESRD).

### Subjects and methods

#### Study design and participants

This cross-sectional case–control study was conducted on 300 patients with type 2 diabetes (T2DM) and 100 healthy control subjects. Patients were selected from outpatients’ Clinic of National Institute for Diabetes and Endocrinology (NIDE), Cairo, Egypt and clinically diagnosed by the NIDE’s professionals according to American Diabetes Association^9^.

*Inclusion and* exclusion *criteria for sample selection*: Patients are clinically diagnosed with T2DM with an age range of 35–65 years, A1C level ≥ 6.5% and diabetes duration > 3 years. All patients were taking the same oral antidiabetic therapy and if hypertension applies, the same antihypertensive drug; angiotensin-converting enzyme Inhibitor (ACEI). For eligible patient cases, DN was defined as a positive persistent urinary albumin excretion “albuminuria” [Urinary Albumin to Creatinine Ratio (UACR) > 30 mg/gm]. All cases with a history of chronic kidney diseases other than DN, those with urinary tract infection, malignancy, liver diseases as well as clinically diagnosed type 1 DM cases were totally excluded from the study cohort. Healthy control subjects were selected from age-, sex- matched volunteers without any chronic or metabolic diseases.

#### Ethics, consent and permissions

Demographic data was recorded for each participant using a self-designed questionnaire. All sample collection procedures were in accordance with the declaration of Helsinki. Written informed consent was obtained from all participants prior to their inclusion in the study. Ethical approval was obtained by the Research Ethics Committee of the General Organization for Teaching Hospitals and Institutes, Cairo, Egypt (Approval No. IDE00276).

The study included 2 main groups: *Group 1*: Healthy subjects’ group (No = 100) and *Group 2*: type 2 diabetic patients (No = 300). For achieving the study goal, diabetic patients were further subclassified, by a specialist, according to their persistent urinary albumin excretion into 2 categories: *DN-confirmed cases*; those having persistent albuminuria, UACR > 30 mg/gm^1^, and *normoalbuminuric diabetic cases;* those having normal albumin excretion, UACR < 30 mg/gm, without any other evidence of any renal pathological changes.

For all subjects, the following variables were also estimated and recorded: age, sex, duration of diabetes, BMI, blood pressure (BP), GFR, UACR and HOMA–IR. BMI was calculated as weight (kg) divided by height squared meter (Kg/m^2^)^10^. Blood pressure (BP) measurements were performed by trained technicians or nurses with a mercury sphygmomanometer and the first and fifth Korotk off sounds were recorded to represent the systolic and diastolic pressure (SBP and DBP), two measurements were obtained and averaged. Hypertension was considered if the SBP was > G130 mm Hg and DBP > G90 mm Hg or if the patient was under hypertension-controlled protocol; only ACEI therapies are of interest. The GFR can be easily estimated using the newer prediction equation developed^11^. Most notably, it is more typical to compare the amount of albumin in the urine sample against its concentration of creatinine. This is termed the UACR expressed in *‘mg/gm* Creatinine’. The use of UACR for these random samples might replace 24-hr urine collections, thus UACR may be a useful diagnostic tool than microalbumin (µalb) itself^12^. UACR was estimated by dividing the µalb. concentration in *mg/L* over the urine Creatinine concentration in *g/L*. UACR was thus expressed in *mg/gm* creatinine. Evaluation of insulin resistance (IR) status was done using the homeostasis model of assessment (HOMA-IR) using the formula: [FPG (mg/dl) × fasting insulin (µIU/ml))/405].

### Methods

Blood samples were collected after overnight fasting from healthy subjects and diabetic patients into three types of vacutainers tubes. The first tube is a serum-separating without any additive in which blood was centrifuged at 3000 rpm for 10 min. Serum was separated and divided into several aliquots; one aliquot was used for fresh measurement of lipid profile, serum creatinine and serum urea while the other aliquots were stored at -80° C for the determination of serum insulin, KNG-1, HSPG, Robo4. The second part of collected blood was taken on EDTA-containing vacutainers for determination of glycosylated hemoglobin (A1C) level; hemolyzed samples were excluded. The third part of collected blood was taken on fluoride-containing vacutainers for determination of Plasma glucose level instantly utilizing glucose oxidase method^13^. Serum total cholesterol was determined by the enzymatic method^14^. Triacylglycerol was assayed by peroxidase-coupled method^15^. HDL-c was measured by enzymatic method^16^. LDL-c was measuring according to Friedewald, et al.^17^. Serum urea was assayed by the enzymatic method according to Tiffany, et al.^18^. Serum creatinine was measured by colorimetric method according to Vasiliades^19^. Sampling, reagent delivery, mixing, processing, calculating and printing were full automatically performed by BT3500 chemistry system (Biotecnica, Instruments Inc, ITALY), and A1C was assayed using ion exchange high performance liquid chromatography (HPLC) on G8 analyzer (Tosoh, Tokyo, Japan). Fresh morning urine sample was collected from each participant into sterile container which used for determination of Microalbumin by immuno-turbidimetric method according to Gentilini, et al.^20^ and urinary creatinine according to Vasiliades^19^. Estimation of UACR was according to Justesen, Petersen, Ekbom, Damm and Mathiesen^12^. Microalbumin measurement and UACR calculation was performed using automated analyzers under standardized laboratory conditions with internal and external quality control. To ensure reliability, all patients were tested on three separate occasions on different days, and the average value was calculated. Microalbuminuria was confirmed based on repeated measurements. Serum insulin was determined using an ELISA kit (DRG International, Inc., USA) according to the manufacturer´s procedure.

Serum KNG-1, HSPG and Robo4 were measured using commercially available ELISA Kits (Catalog numbers: In-Hu2714, In-Hu4252 and In-Hu1403 respectively - INNOVA BIOTECH, CHINA) according to the manufacturer´s procedure.

### Statistical analysis

All statistical analyses were performed using IBM SPSS Statistics for Windows, Version 22.0 (IBM Corp., Armonk, NY, USA; https://www.ibm.com/products/spss-statistics). The Kolmogorov–Smirnov test was applied to verify the normality of continuous variables. Whenever applicable, data were expressed as mean ± standard error of the mean (M ± SEM).

Comparisons between two independent groups were conducted using the unpaired Student’s t-test, and Pearson’s correlation was used to assess linear associations between continuous variables. To evaluate the combined effects of hypertension and DN on serum biomarkers, two-way ANOVA was applied. Chi-square test was used for categorical variable comparisons. Logistic regression analysis was employed to confirm chi-square test exploring the impact of hypertension on the progression of DN, while adjusting for potential confounding factors, including demographic and metabolic covariates.

The Receiver Operating Characteristic (ROC) curve analysis was utilized to assess the diagnostic performance of individual biomarkers, with area under the curve (AUC) values reported to determine sensitivity and specificity. A p-value < 0.05 was considered statistically significant.

To further address the incremental clinical value of the investigated biomarkers beyond traditional indicators of diabetic nephropathy, additional analyses were performed. First, subgroup analyses were conducted within normoalbuminuric subjects (UACR < 30 mg/g creatinine) to explore the potential of KNG-1, HSPG, and Robo4 in association with early renal functional alterations prior to overt albuminuria. Within this subgroup, Pearson correlation and multivariable linear regression analyses were performed using eGFR as a surrogate marker of renal function decline.

Second, to evaluate the incremental predictive value of the biomarkers, multivariable logistic regression models were constructed with albuminuria status as the dependent variable. A baseline model including clinical and metabolic variables was first established, followed by a second model incorporating the studied biomarkers. Model performance was compared using − 2 Log Likelihood, Nagelkerke R², and ROC curve analysis based on predicted probabilities derived from each model. This approach was used to assess whether the inclusion of KNG-1, HSPG, and Robo4 improves risk stratification beyond conventional clinical parameters.

## Results

This study was performed on 300 (200 with DN and 100 without) outpatients with T2DM (Disease duration: 14.23 ± 0.38 years) and 100 healthy subjects as a control group. Different parameters were investigated and correlated in different disease situations. For accurate comparison, healthy subjects were selected with comparable non-significant (*p* > 0.05) age and sex distributions compared to diabetic subjects (55.17 ± 1.05 vs. 55.43 ± 0.53 years; 40 males & 60 females vs. 120 males & 180 females).

Table 1 displays the participants’ demographic and biochemical characteristics. As described, a significant difference (P ˂ 0.05) was found in BMI, SBP, DBP, FPG, A1C, HOMA-IR, cholesterol, TG, HDL, urea, creatinine, UACR, GFR, HSPG and Robo4 when compared to healthy subjects. In contrast, KNG-1 serum level failed to show significant change between diabetics and controls.

Among diabetic patients, Table 2 summarizes demographic and biochemical characteristics of patients diagnosed with DN (Persistent Albuminuria; No = 100) and those without (Normoalbuminuria; No = 200). Again, participants of those 2 subgroups were preselected in age-, sex- and BMI-matched distribution manner (*P* > 0.05) for precise comparison. As shown in the table, no statistical significance was found regarding HOMA-IR, HDL-c, LDL-c, urea, creatinine, GFR (*P* > 0.05) while SBP, DBP, FBG, A1C, Cholesterol, TG, UACR, KNG1, HSPG and Robo4 were changed dramatically when the 2 subgroups were compared.

To study the impact of hypertension on diabetic patients’ cohorts with and without DN, we did further classification to patients according to having hypertension. Hypertensive diabetic patients with and without DN were compared to normotensive homolog groups. As illustrated in Table 3, the three investigated parameters KNG-1, HSPG and Robo4 were approximately duplicated (P ˂ 0.05) in their concentrations when all hypertensive patients (No = 140) were compared with normotensives (No = 160). Surprisingly, the same was applied to microalbumin concentration which shows about 3-fold-change (P ˂ 0.05) in hypertensive diabetic patients. Notably, the same pattern of those 4 biomarkers was further intensified (P ˂ 0.05) in DN hypertensive patients when compared to DN normotensive cohort. On the other hand, no effect (*P* > 0.05) was observed in Normoalbuminuric diabetic patients with and without hypertension regarding those biomarker profiles. All other characteristics and investigated parameters did not change significantly (*P* > 0.05) when hypertensive patients with or without DN were compared with corresponding normotensive cohorts except for creatinine (P ˂ 0.05).

To show how the profile pattern of KNG-1, HSPG and Robo4 are correlated with other biochemical data of participants, parametric linear correlation testing in-between the investigated parameters were performed with results organized in Table (4). Interestingly, the profile pattern of the 3 parameter levels showed moderate to strong positive correlations (P ˂ 0.01) with both BP and albuminuria. In addition, strong positive cross-correlations (P ˂ 0.01) in-between profile patterns of KNG-1, HSPG and Robo4 were also noticeable.

**Table 1:** **Demographic and biochemical characteristics of main studied cohorts:**

Parameters	Healthy subjects (No = 100)	Type 2 diabetic patients (No = 300)
Age (year)	55.17 ± 1.05	55.43 ± 0.53
Disease Duration (years)	-----------------------	14.23 ± 0.38*
BMI (Kg/m^2^)	28.6 ± 0.58	32.92 ± 0.36*
SEX (M/F)	40/60	120/180
SBP (mmHg)	117.62 ± 0.65	133.21 ± 0.76*
DBP (mmHg)	77.17 ± 0.48	87.27 ± 0.52*
FPG (mg/dl)	93.0 ± 0.76	243.67 ± 6.6*
A1C (%)	5.34 ± 0.03	9.30 ± 0.13*
HOMA-IR	1.86 ± 0.11	10.33 ± 0.6*
Cholesterol (mg/dl)	196.46 ± 3.9	213.27 ± 3.97*
TG (mg/dl)	139.05 ± 7.25	202.43 ± 8.46*
HDL-c (mg/dl)	45.33 ± 1.35	42.16 ± 0.74*
LDL-c (mg/dl)	123.32 ± 3.08	130.62 ± 3.36
Urea (mg/dl)	31.38 ± 0.98	38.15 ± 1.01*
Creatinine (mg/dl)	0.95 ± 0.02	1.15 ± 0.03*
UACR (mg/gm)	7.04 ± 0.68	343.18 ± 31.87*
GFR (ml/min/1.73m^2^)	82.58 ± 1.73	68.88 ± 1.26*
KNG-1(pg/ml)	4769.56 ± 348.9	4534.89 ± 183.02
HSPG (pg/ml)	142.53 ± 8.06	208.40 ± 8.45*
Robo4 (pg/ml)	1643.33 ± 91.28	1922.74 ± 73.36*

All data are expressed as the M ± SEM and approximated to the second digit.

Significant data are shown in **bold**. *: *p* < 0.05 compared to the Healthy control group using independent samples t-student test.

BMI: Body mass index, BP: blood pressure; FPG: Fasting plasma glucose; A1C: Glycated hemoglobin; HOMA-IR: Homeostatic model assessment for insulin resistance; TG: Triglycerides; HDL-c: High density lipoprotein cholesterol; LDL-c: Low density lipoprotein cholesterol; UACR: Albumin Creatinine ratio; GFR: Glomerular filtration rate; KNG-1: Kininogen-1; HSPG: Basement membrane specific Heparan sulfate proteoglycan; Robo4: Roundabout homolog 4.

**Table 2:** **Demographic and biochemical characteristics of type 2 diabetic patients with and without DN (Persistent albuminuria):.**

Parameters	Diabetic patients with Normoalbuminuria (No = 100)	Diabetic patients with Albuminuria (No = 200)
Age (year)	55.42 ± 0.96	55.45 ± 0.64
Disease Duration (years)	12.65 ± 0.63	15.02 ± 0.46*
BMI (Kg/m^2^)	33.7 ± 0.63	32.52 ± 0.43
SEX (M/F)	40/60	80/120
SBP (mmHg)	127.1 ± 1.08	136.28 ± 0.92*
DBP (mmHg)	71.12 ± 2.27	67.75 ± 1.51*
FPG (mg/dl)	217.21 ± 10.48	256.91 ± 8.25*
A1C (%)	8.52 ± 0.19	9.70 ± 0.16*
HOMA-IR	9.31 ± 0.83	10.55 ± 0.79
Cholesterol (mg/dl)	200.66 ± 4.90	219.57 ± 5.37*
TG (mg/dl)	175.65 ± 11.43	215.82 ± 11.22*
HDL-c (mg/dl)	40.63 ± 1.29	42.93 ± 0.89
LDL-c (mg/dl)	124.9 ± 4.31	133.47 ± 4.55
Urea (mg/dl)	39.95 ± 1.71	37.26 ± 1.24
Creatinine (mg/dl)	1.13 ± 0.03	1.16 ± 0.03
UACR (mg/gm)	9.61 ± 0.73	509.9 ± 43.24*
GFR (ml/min/1.73m^2^)	71.12 ± 2.27	67.75 ± 1.51
KNG-1(pg/ml)	1936.04 ± 76.7	5834.32 ± 220.4*
HSPG (pg/ml)	121.19 ± 6.58	252.01 ± 11.03*
Robo4 (pg/ml)	1105.4 ± 46.28	2331.41 ± 95.29*

All data are expressed as the *M ± SEM* and approximated to the second digit.

Significant data are shown in **bold**. *: *p* < 0.05 compared to the Healthy control group using independent samples t-student test.

Normoalbuminuria was defined as any case with UACR ˂ 30 mg/gm creatinine while albuminuria was defined as any case with persistent UACR > 30 mg/gm creatinine (Cases diagnosed with DN).

BMI: Body mass index, BP: blood pressure; FPG: Fasting plasma glucose; A1C: Glycated hemoglobin; HOMA-IR: Homeostatic model assessment for insulin resistance; TG: Triglycerides; HDL-c: High density lipoprotein cholesterol; LDL-c: Low density lipoprotein cholesterol; UACR: Albumin Creatinine ratio; GFR: Glomerular filtration rate; KNG-1: Kininogen-1; HSPG: Basement membrane specific Heparan sulfate proteoglycan; Robo4: Roundabout homolog 4.

**Table 3:** **Impact of hypertension on diabetic patients’ cohorts with and without DN.**

Parameter	Normotensive diabetic patients			Hypertensive Diabetic Patients		
Number	Total cohort (No = 160)	Normo-albuminuria (No = 72)	Albuminuria (No = 88)	Total cohort (No = 140)	Normo-albuminuria (No = 28)	Albuminuria (No = 112)
Age (year)	54.82 ± 0.76	53.92 ± 1.17	55.57 ± 1.00	56.14 ± 0.74	59.29 ± 1.38*	55.35 ± 0.85
Disease Duration (years)	14.1 ± 0.56	12.07 ± 0.78	15.76 ± 0.76	14.38 ± 0.5	14.14 ± 1.08	14.44 ± 0.57
BMI (Kg/m^2^)	33.12 ± 0.53	33.02 ± 0.77	33.2 ± 0.73	32.69 ± 0.47	35.47 ± 1.0	32.0 ± 0.51
SBP (mmHg)	123.06 ± 0.54	121.67 ± 0.84	124.2 ± 0.68	144.82 ± 0.67*	141.07 ± 0.79*	145.76 ± 0.79*
DBP (mmHg)	81.56 ± 0.51	80.69 ± 0.82	82.27 ± 0.64	93.79 ± 0.59*	90.36 ± 1.41*	94.64 ± 0.62*
FPG (mg/dl)	247.2 ± 9.55	216.11 ± 12.05	272.64 ± 13.76	239.64 ± 9.02	220.04 ± 21.36	244.54 ± 9.92
A1C (%)	9.27 ± 0.18	8.54 ± 0.23	9.88 ± 0.21	9.35 ± 0.2	8.48 ± 0.34	9.57 ± 0.23
HOMA-IR	9.97 ± 0.78	8.82 ± 0.92	10.91 ± 1.2	10.35 ± 0.92	10.59 ± 1.8	10.28 ± 1.06
Cholesterol (mg/dl)	216.38 ± ± 5.4	199.93 ± 6.1	229.84 ± 8.21	209.71 ± 5.86	202.54 ± 8.16	211.5 ± 7.04
TG (mg/dl)	210.4 ± 11.16	181.93 ± 15.04	233.81 ± 15.79	193.24 ± 12.87	159.5 ± 13.01	201.68 ± 15.68
HDL-c (mg/dl)	42.44 ± 1.06	40.28 ± 1.52	44.22 ± 1.46	41.84 ± 1.01	41.54 ± 2.51	41.92 ± 1.1
LDL-c (mg/dl)	131.84 ± 4.81	123.27 ± 5.43	138.86 ± 7.49	129.22 ± 4.67	129.1 ± 6.51	129.24 ± 5.62
Urea (mg/dl)	37.91 ± 1.29	37.47 ± 1.6	38.36 ± 1.95	38.37 ± 1.59	46.32 ± 4.37	36.38 ± 1.62
Creatinine (mg/dl)	1.14 ± 0.04	1.08 ± 0.04	1.19 ± 0.06	1.16 ± 0.03	1.26 ± 0.07*	1.13 ± 0.04
UACR (mg/gm)	177.62 ± 27.16	9.61 ± 0.85	315.08 ± 44.35	532.39 ± 56.88*	9.61 ± 1.47	663.08 ± 65.53*
GFR (ml/min/1.73m^2^)	70.08 ± 1.68	73.52 ± 2.63	67.27 ± 2.14	67.5 ± 1.91	64.97 ± 4.36	68.13 ± 2.12
KNG-1 (pg/ml)	3077.36 ± 180.78	2016.67 ± 94.13	3945.19 ± 288.87	6200.66 ± 272.4*	1728.72 ± 122.16	7318.64 ± 242.93*
HSPG (pg/ml)	165.65 ± 8.14	124.07 ± 7.73	199.67 ± 12.27	257.28 ± 14.52*	113.8 ± 12.66	293.15 ± 16.2*
Robo4 (pg/ml)	1396.56 ± 84.81	1132.25 ± 54.32	1612.81 ± 143.99	2524.1 ± 102.62*	1036.35 ± 88.6	2896.04 ± 98.87*

All data are expressed as the *M ± SEM* and approximated to the second digit.

Significant data are shown in **bold**. *: *p* < 0.05 compared to the Corresponding Normotensive Cohort using independent samples t-student test.

Hypertensive cohort is defined as those cases with BP > 130/90 while normotensive cohorts are those cases with lower BP values.

Normoalbuminuria was defined as any case with UACR ˂ 30 mg/gm creatinine while albuminuria was defined as any case with persistent UACR > 30 mg/gm creatinine (Cases diagnosed with DN).

BMI: Body mass index, BP: blood pressure; FPG: Fasting plasma glucose; A1C: Glycated hemoglobin; HOMA-IR: Homeostatic model assessment for insulin resistance; TG: Triglycerides; HDL-c: High density lipoprotein cholesterol; LDL-c: Low density lipoprotein cholesterol; UACR: Albumin Creatinine ratio; GFR: Glomerular filtration rate; KNG-1: Kininogen-1; HSPG: Basement membrane specific Heparan sulfate proteoglycan; Robo4: Roundabout homolog 4.

**Table 4:** **Parametric linear correlations in-between the investigated parameters.**

Comparison			All study cohorts (No = 400)	Diabetic cohort (No = 300)
			*r*	*r*
KNG-1	Vs.	BMI	------------	− 0.129*
		SBP	0.460**	0.638**
		DBP	0.330**	0.493**
		A1C	------------	0.117*
		GFR	− 0.103*	------------
		s. creatinine	0.107*	------------
		UACR	0.526**	0.663**
HSPG	Vs.	Duration of diabetes	0.22**	------------
		SBP	0.443**	0.433**
		DBP	0.396**	0.387**
		GFR	− 0.120*	------------
		FPG	0.175**	------------
		A1C	0.197**	------------
		Urea	0.129*	------------
		s. creatinine	0.207**	0.182**
		UACR	0.698**	0.714**
		KNG-1	0.631**	0.720**
ROBO4	Vs.	BMI	------------	0.137*
		Duration of diabetes	0.137**	------------
		SBP	0.394**	0.450**
		DBP	0.321**	0.374**
		A1C	0.124*	------------
		UACR	0.447**	0.475**
		KNG-1	0.609**	0.687**
		HSPG	0.497**	0.484**

Significant data are shown in **bold** while insignificant data are ignored. *, **: *p* < 0.05, *p* < 0.01 respectively using Pearson linear correlation analysis. A *negative* sign indicates *inverse linear correlation*. (**r)**: Pearson rank correlation coefficient assuming Gaussian distributions.

BMI: Body mass index, BP: blood pressure; FPG: Fasting plasma glucose; A1C: Glycated hemoglobin; GFR: Glomerular Filtration Rate, UACR: Urinary Albumin Creatinine ratio; KNG-1: Kininogen-1; HSPG: Basement membrane specific Heparan sulfate proteoglycan; Robo4: Roundabout homolog 4.

Up to now, results have guided us to a suggestive collaborative combined influence of both hypertension and albuminuria on the profile patterns of KNG-1, HSPG and Robo4 in diabetic patients. This targeted us to postulate 2 new aims for this study; firstly, to study how the presence of hypertension, as contributing factor, could *independently* influence the development (susceptibility) of DN progression “persistent albuminuria” as a complication in T2DM. Secondly, to study how the combined effect of both Hypertension and Albuminuria *together* could impact the expression profile pattern of the investigated biomarkers. In other words, we would like to understand the interaction between both Hypertension and Albuminuria in modulating the profile data of KNG-1, HSPG and Robo4.

In order to achieve the first aim, first, a chi square test for independence was adopted, results showed that the presence of hypertension among diabetic cohort *independently* accelerates the development of DN at *p* < 0.000. To ensure and confirm results, logistic regression analysis was followed with adjustment of other contributing covariates (gender, BMI, disease duration, A1C and TGs levels). After adjustment, results came supporting chi square data; hypertension remained a significant independent predictor of albuminuria, indicating that its association with DN is robust and not explained by these clinical covariates (P_adj_. < 0.000).

**Fig. 1:**
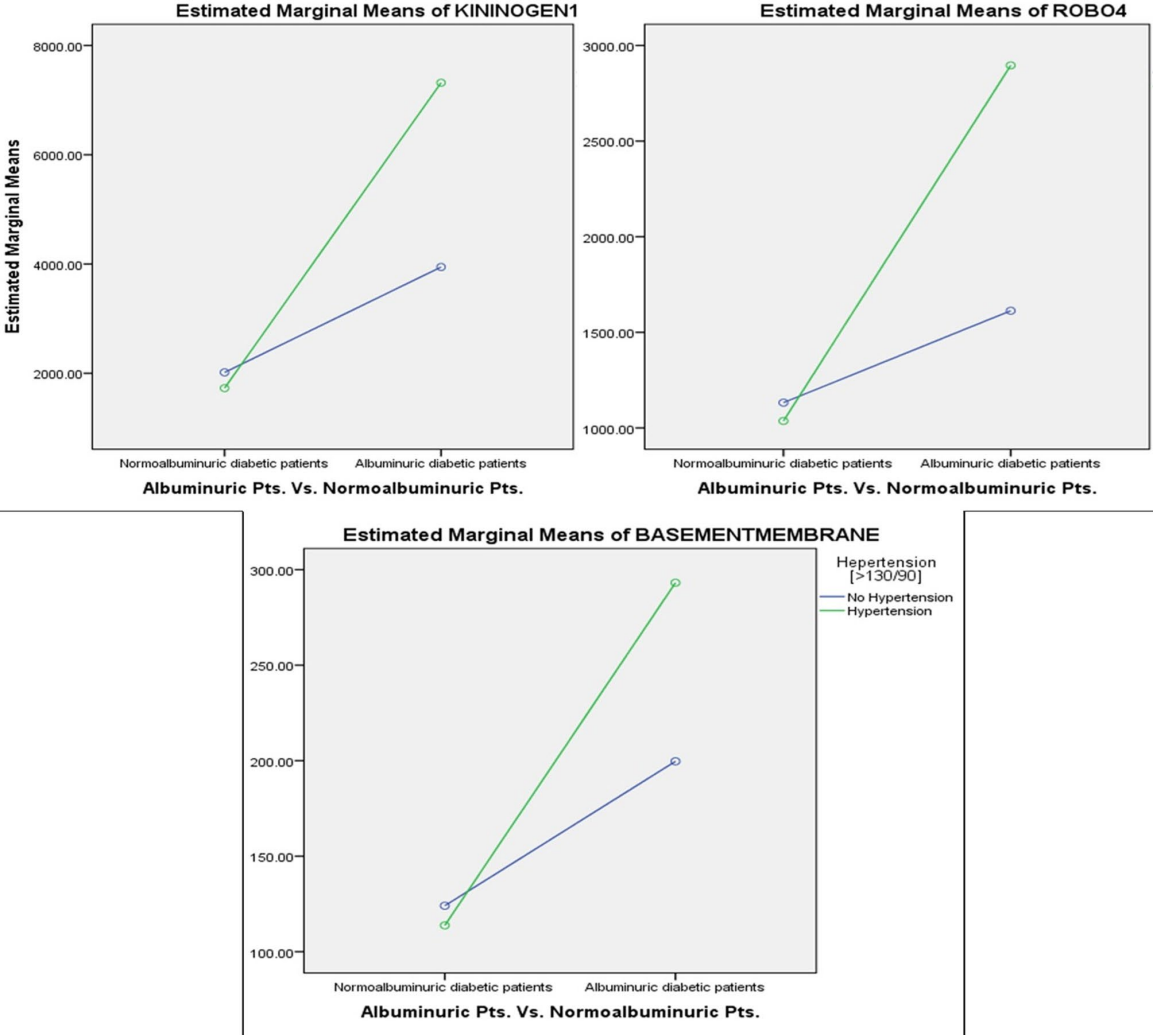
**Profile plots for the interaction of both Hypertension and Albuminuria statuses on the expression levels of the three studied biomarkers (KNG-1, HSPG and ROBO4). **Data - expressed as estimated marginal means - were verified and emerged using two-way ANOVA analysis; the three parameters showed *divergence* indicating synergistic effect on DN susceptibility beyond each independent factor. P-value and Partial Eta Squared results were shown in Table 5.

For the second aim to be achieved, the effects of hypertension status, albuminuria status, and their interaction on the expression levels of the three studied biomarkers were intensively analyzed using two-way ANOVA (Table 5). This type of analysis facilitates investigating the combined impact of both Hypertension and Albuminuria as well as understanding the interaction between them in modulating the expression profiles of KNG-1, HSPG and Robo4. Significant interaction effects were detected for all biomarkers, as illustrated by the *divergence* observed in the profile plots (Fig. 1). For KNG-1, both hypertension status (*p* < 0.001, partial η² = 0.087) and albuminuria status (*p* < 0.001, partial η² = 0.361) had significant *independent* effects. Combined, a significant *interaction* between hypertension and albuminuria was found (*p* < 0.001, partial η² = 0.118). Regarding HSPG, hypertension status (*p* = 0.014, partial η² = 0.020) and albuminuria status (*p* < 0.001, partial η² = 0.161) *independently* affected biomarker levels. Together, the *interaction* between hypertension and albuminuria was also significant (*p* = 0.002, partial η² = 0.031). For ROBO4, hypertension status showed a particularly strong *independent* effect (*p* < 0.001, partial η² = 0.630), as did albuminuria status (*p* < 0.001, partial η² = 0.206). Their interaction effect was also statistically significant (*p* = 0.002, partial η² = 0.083).

**Table 5:** **Two-Way ANOVA results showing the independent and interaction effects of hypertension and Albuminuria on investigated biomarkers (KNG-1, HSPG and ROBO4).**

Biomarker	Effect	Source	F-value	*P*-value	Partial Eta Squared (partial η²)
KNG-1	1	Hypertension status	28.212	*P* < 0.001	0.087
	2	Albuminuria status	167.507	*P* < 0.001	0.361
	3	Hypertension* albuminuria status	39.725	*P* < 0.001	0.118
HSPG	1	Hypertension status	6.056	*P* < 0.05	0.02
	2	Albuminuria status	56.858	*P* < 0.001	0.161
	3	Hypertension* albuminuria status	9.417	*P* < 0.01	0.031
ROBO4	1	Hypertension status	19.816	*P* < 0.001	0.063
	2	Albuminuria status	76.986	*P* < 0.001	0.206
	3	Hypertension* albuminuria status	26.736	*P* < 0.001	0.083

• Significant data are shown in P-value column.

• 1, 2: Show the effect of each *independent* factor alone after *adjusting* the other. 3: Shows the combined effect of the 2 factors after applying their *interaction*.

• F-value measures the strength of the effect (higher F = stronger evidence against the null hypothesis).

• Partial Eta Squared “η²”: is a measure of effect size used in ANOVA. It measures “how big” the impact of each factor is, showing the strength and importance of each independent variable (after adjusting others) and their interaction on your biomarker levels.

**Fig. 2:**
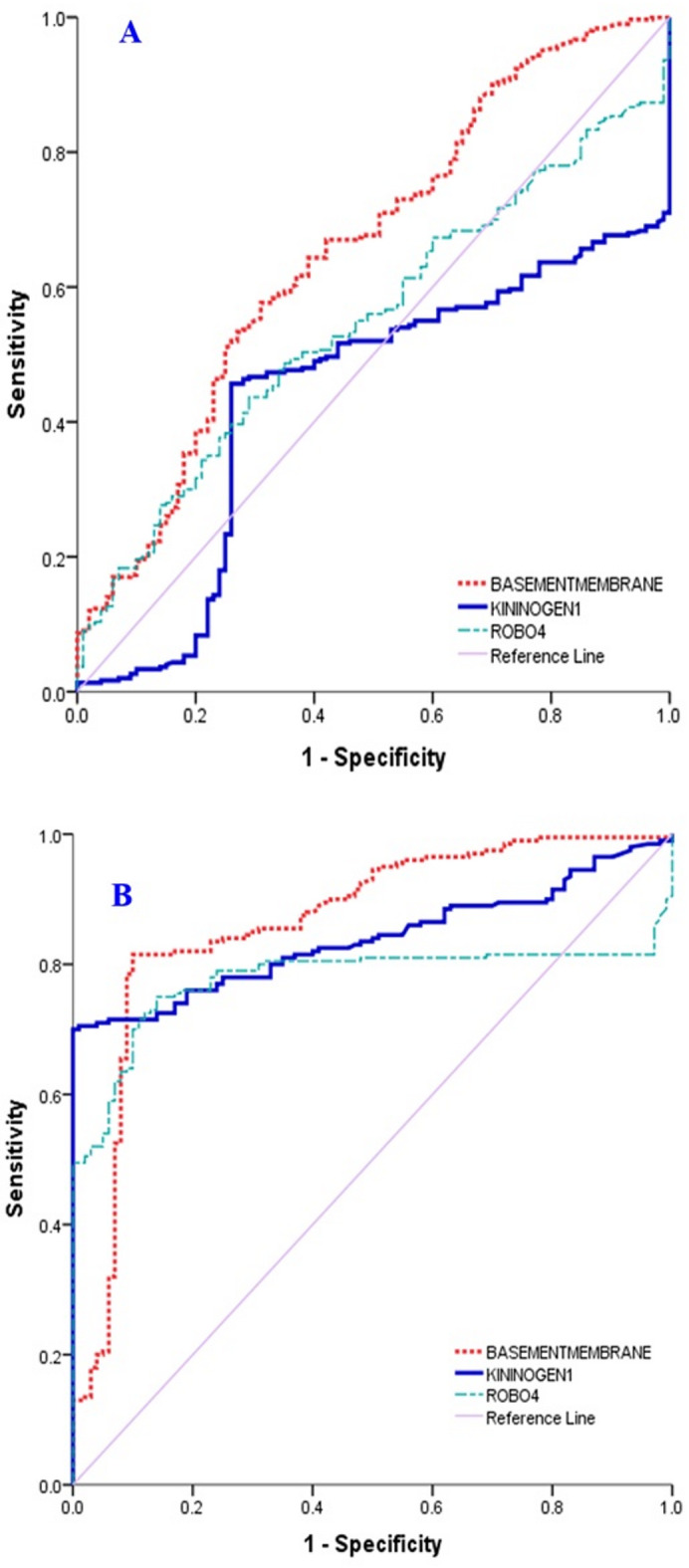
**ROC curves of KNG-1, HSPG and Robo4.** The three investigated parameters were compared and tested for their discriminative ability in diagnosing 2 clinical situations: Diabetic vs. Healthy subjects (Fig. 2A) as well as Albuminuric vs. Non-albuminuric patients (Fig. 2B). Data - expressed as AUC - were verified using ROC analysis. P-value and AUC results were shown in Table 6. Figure (2A): Among the three investigated biomarkers, HSPG showed *fair* discrimination, while KNG-1 and Robo4 demonstrated *poor* diagnostic utility in detecting diabetes itself. Figure (2B): All biomarkers demonstrated *strong* diagnostic accuracy, with HSPG and KNG-1 showing excellent ability to differentiate albuminuric from non-albuminuric diabetic individuals. HSPG was the closest to the top left-hand corner showing the highest accuracy and the biggest AUC (≈ 0.86). KNG-1, then Robo4 came in descending order of accuracy and AUC.

As represented in Fig. 2 and Table 6, ROC (Receiver Operating Characteristic) analysis was performed in order to evaluate the diagnostic performance for the three biomarkers: KNG-1, HSPG and Robo4 under two clinical comparisons. First, for KNG-1, when diabetic patients were compared vs. healthy subjects (No = 400), (AUC = 0.436; *P* = 0.056), results indicate that KNG-1 is not useful in differentiating diabetics from healthy subjects. However, when tested for differentiating between type 2 diabetic patients with DN and those without DN (No = 300), (AUC = 0.839; *P* = 0.000), results of KNG-1 showed excellent discriminatory ability. Second, regarding HSPG, when diabetic patients were compared vs. healthy subjects (No = 400), (AUC = 0.657; *P* = 0.000), results indicate that HSPG have fair diagnostic utility. But in case of the type 2 diabetic patients with DN, when compared vs. those without DN (No = 300), (AUC = 0.864; *P* = 0.000), results of HSPG showed excellent capability to distinguish between them. In the same context, Robo4 exhibits no capability to discriminate diabetic patients from healthy subjects (No = 400), (AUC = 0.547; *P* = 0.161). While, it appears to have good diagnostic ability in discriminating DN patients form all diabetic subjects (No = 300), (AUC = 0.777; *P* = 0.000).

**Table 6:** **ROC analysis evaluating the diagnostic performance for the three biomarkers: KNG-1, HSPG and Robo4 under two clinical comparisons;** *Diabetic vs. Healthy subjects* (Fig. 2A) as well as *Albuminuric vs. Non-albuminuric patients* (Fig. 2B).

Parameter	Groups (Clinical comparisons)		No	AUC	*P*-Value
KNG-1	1	Healthy vs. diabetic patients	400	0.436	*P* > 0.05
	2	Albuminuric vs. Non-albuminuric patients	300	0.839	*P* < 0.001
HSPG	1	Healthy vs. diabetic patients	400	0.657	*P* < 0.001
	2	Albuminuric vs. Non-albuminuric patients	300	0.864	*P* < 0.001
Robo4	1	Healthy vs. diabetic patients	400	0.547	*P* > 0.05
	2	Albuminuric vs. Non-albuminuric patients	300	0.777	*P* < 0.001

Significant P-Values are shown indicating *discriminative* ability of the corresponding biomarker. AUC: Area under the curve; the bigger AUC value, the higher the overall accuracy of the corresponding biomarker with respect to its discriminative ability.

To further investigate the potential clinical relevance of the studied biomarkers beyond albuminuria, several additional analyses were performed. First, *within the normoalbuminuric subgroup*, several analyses were performed including linear correlations, multivariable Linear Regression, as well as within-normal UACR subgroup stratification analyses.

Bivariate linear correlation analysis revealed that Robo4 was modestly but significantly correlated with GFR (*r* = 0.196, *p* < 0.01), while all biomarkers demonstrated significant associations with blood pressure parameters (SBP, DBP) (Table 7).

Also, among normoalbuminuric subjects, we performed Multivariable Linear Regression analysis models with GFR as Dependent variable: Model 1 include the following independent variables: Age, Sex, BMI, A1C, SBP, UACR. Model 2, we added the investigated biomarkers (KNG-1, HSPG, ROBO4) in order to evaluate their independent early association with renal function decline while other independent factors are adjusted. R square and Beta (standardized) were reported. the inclusion of KNG-1, HSPG, and Robo4 resulted in a modest increase in the explained variance of GFR (R² increased from 0.133 to 0.157), with Robo4 emerging as a borderline independent predictor (*p* = 0.05) (Table 8).

In order to test whether biomarkers can detect subtle risk even when UACR is “normal”, we considered within-normal UACR subgroup stratification. We divided normoalbuminuric subjects into 3 subgroups; Low-normal UACR (< 10 mg/g), Middle-normal UACR (10–20 mg/g) and High-normal UACR (20–30 mg/g) then we compared biomarkers in-between each 2 subgroups using independent sample t-test in order to test whether biomarkers can stratify normoalbuminuric subjects which may support early detection. Unfortunately, results may not reflect reality due to low number of normoalbuminuric subjects who were further subdivided into 3 subgroups (Table 9).

**Fig. 3:**
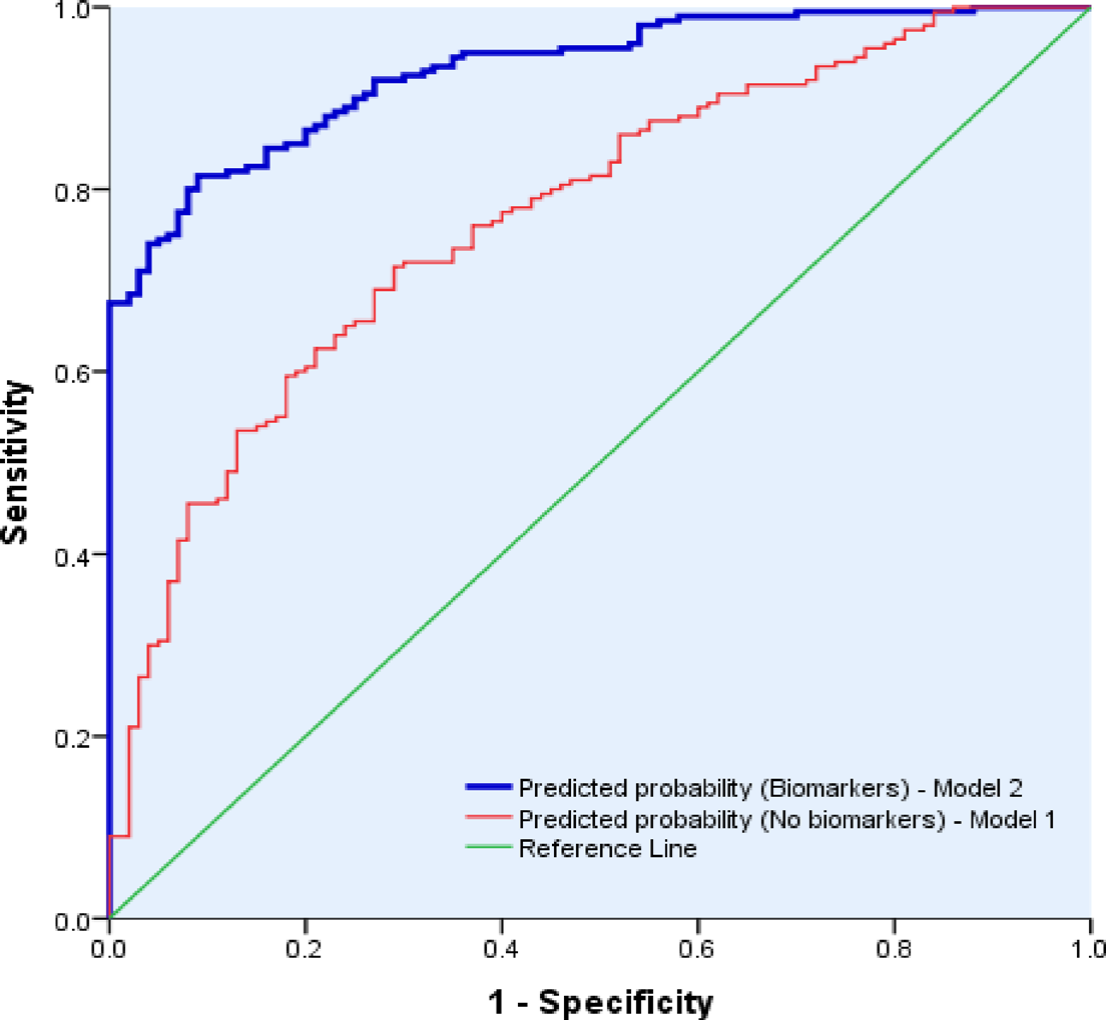
**ROC curve illustrating the discriminative performance of predicted probabilities obtained from multivariable logistic regression models.** Model 1 is based on clinical variables only (Age, Sex, BMI, disease duration A1C, SBP.), whereas Model 2 incorporates clinical variables along with the biomarkers KNG-1, HSPG, and Robo4. The addition of biomarkers resulted in a marked improvement in model discrimination, with the AUC increasing from 0.771 to 0.931.

Second, *among all T2DM patients*, in order to address the incremental value of the biomarkers in predicting renal risk, we performed multivariable logistic regression analysis models with Albuminuria status (yes or no) as Dependent variable: Model 1 include the following independent clinical variables: Age, Sex, BMI, disease duration A1C, SBP. Notably, since albuminuria was used as the defining outcome, UACR was not included as a predictor. Model 2, we added the investigated biomarkers (KNG-1, HSPG, ROBO4) in order to evaluate their independent prediction of renal involvement risk while other independent clinical factors are adjusted. We saved the calculated predicted probabilities for both models in order to perform ROC analysis discriminating between the 2 models and comparing the AUC in both situations. We reported p-values with their Odds Ratio (Exp(B)), model-fit statistics (-2 Log Likelihood and Nagelkerke R²) and AUC. Analysis demonstrated that the addition of KNG-1, HSPG, and Robo4 to clinical variables markedly improved model performance. Specifically, Nagelkerke R² increased from 0.279 in the clinical model to 0.665 after inclusion of biomarkers, accompanied by a substantial reduction in -2 Log Likelihood (Table 10). ROCcurve analysis confirmed a significant improvement in discriminative ability, with AUC increasing from 0.771 to 0.931 (Table 10; Fig. 3). These findings indicate that the investigated biomarkers may provide significant incremental predictive value beyond conventional clinical parameters in identifying renal involvement in T2DM patients.

**Table 7:** **Pairwise linear correlations in-between the investigated parameters among normoalbuminuric subjects.**

Comparison			Normoalbuminuric cohort
			r
KNG-1	Vs.	SBP	- 0.163*
		DBP	- 0.245**
		GFR	0.055
		HSPG	0.404**
		ROBO4	0.458**
HSPG	Vs.	SBP	-0.055
		DBP	-0.069
		GFR	0.081
		ROBO4	0.527**
ROBO4	Vs.	SBP	- 0.199**
		DBP	- 0.250**
		GFR	0.196**

Significant data are shown in **bold.** *, **: P-value < 0.05 and 0.01 respectively using Pearson linear correlation analysis. A *negative* sign indicates *inverse linear correlation*. (**r)**: Pearson rank correlation coefficient assuming Gaussian distributions.

SBP: systolic blood pressure; DBP: diastolic blood pressure; A1C: Glycated hemoglobin; GFR: Glomerular Filtration Rate, UACR: Urinary Albumin Creatinine ratio; KNG-1: Kininogen-1; HSPG: Basement membrane specific Heparan sulfate proteoglycan; Robo4: Roundabout homolog 4.

**Table 8:** **Multivariable linear regression among normoalbuminuric subjects.**

Independent predictor	Variable significance		Model summary		Dependent variable
	*P*-value	β standardized coefficient	*R*²	Adjusted *R*²	
A. Model 1 (clinical independent variables only): Age, Sex, BMI, A1C, SBP, UACR					
Gender	**< 0.05**	0.164	0.133	0.106	GFR
A1C	**< 0.05**	-0.182			
SBP	**< 0.05**	-0.170			
Age, BMI, UACR	> 0.05	----			
B. Model 2 (clinical independent variables + Biomarkers):					
KNG-1	> 0.05	-0.135	0.157	0.118	GFR
HSPG	> 0.05	-0.012			
Robo4	**= 0.05**	0.169			

**β (Beta): **Standardized regression coefficient indicating the strength and direction of association reflecting the relative contribution of each predictor; **R²: **Represents the proportion of variance in the dependent variable explained by the model; **Adjusted R²: **variance explained by the model adjusted for the number of predictors in the model.

**Table 9:** **Within-normal UACR stratification analysis.**

Parameters	Low-normal UACR (< 10 mg/g)	Middle-normal UACR (10–20 mg/g)	High-normal UACR (20–30 mg/g)
KNG-1(pg/ml)	3608.93 ± 259.97	2625.66 ± 324.95*	3110.44 ± 294.29
HSPG (pg/ml)	133.92 ± 6.32	118.18 ± 10.24	155.39 ± 21.29
Robo4 (pg/ml)	1424.28 ± 67.94	1180.99 ± 96.49*	1493.26 ± 87.03

All data are expressed as the *M ± SEM* and approximated to the second digit.

Significant data are shown in **bold**. *: *p* < 0.05 compared to the Low-normal UACR (< 10 mg/g) subgroup using independent samples t-student test.

KNG-1: Kininogen-1; HSPG: Basement membrane specific Heparan sulfate proteoglycan; Robo4: Roundabout homolog 4.

**Table 10:** **Multivariable logistic regression analysis among T2DM patients.**

Independent variables	Variable significance & odds ratio			Model improvement parameters	ROC data		Dependent variable
	*P*-value	Exp(B)	-2 Log Likelihood	Nagelkerke *R*²	AUC	*P*-value	
Model 1 (clinical independent variables only): Age, Sex, BMI, disease duration A1C, SBP.							
Duration	< 0.01	1.066	314.738	0.279	0.771	< 0.001	Albuminuria status
A1C	< 0.001	1.320					
SBP	< 0.001	1.073					
Age, BMI, Gender	> 0.05	----					
Model 2 (clinical independent variables + Biomarkers):							
KNG-1	< 0.001	1.001	186.510	0.665	0.931	< 0.001	Albuminuria status
HSPG	< 0.01	1.010					
Robo4	< 0.05	0.999					

**Exp(B) (odds ratio): **reflects the change in odds of the outcome per unit increase in the predictor; **−2 Log Likelihood: **indicates overall model fit (lower values denote better fit); **Nagelkerke R²: **represents the proportion of variance in the outcome explained by the model. **AUC**: Area under the curve; the bigger AUC value, the higher the overall accuracy of the corresponding model with respect to its discriminative ability.

## Discussion

This study aimed to explore specific serum proteomic signature profiles of KNG-1, HSPG, and Robo4 that may serve as potential indicators of renal involvement in T2DM patients, particularly in the presence of hypertension. Given that DN often remains clinically silent until significant kidney damage has occurred, identifying early pathologic alterations is crucial for timely intervention. The study also investigated the potential interactive influence of hypertension and DN on these biomarkers, with the goal of improving early diagnostic accuracy.

In this study, approximately 60% of the participants are females, reflecting the predominance of women among the type 2 diabetic population in Egypt. Healthy control subjects were selected to be age- and sex-matched with diabetic patients to ensure accurate comparisons and minimize the potential influence of age and gender differences. However, we were unable to achieve equal BMI distribution between diabetic and control groups. This imbalance likely reflects the high prevalence of obesity as a major risk factor among individuals with T2DM. Nevertheless, there is no current evidence to suggest that differences in BMI significantly affect the parameters investigated in this study.

The same considerations were applied when selecting patients with and without albuminuria to ensure that age and gender were balanced across all comparison groups, allowing for more accurate results. However, BMI adjustment was specifically applied as a selection criterion for patients with and without albuminuria, as obesity is an independent risk factor for the progression of DN among individuals with diabetes. In 2024, Azagew et al.^1^, emphasized that DN is often multifactorial and is associated with factors such as being overweight, smoking, type of diabetes and non-adherence to diabetic medications.

Regarding the duration of the diabetic course, the mean disease duration among type 2 diabetic patients was approximately 14 years, compared to controls. This was intentionally considered during patient selection to ensure sufficient time for the development of diabetic complications, particularly DN. *Vinter-Repalust and his coworkers*^21^ previously reported that a duration of diabetes of 10 years or more significantly increases the risk of developing DN. Naturally, prolonged disease duration, especially when coupled with poor glycemic control, contributes to renal vascular damage and subsequent impairment of kidney function^22^.

Unfortunately, hypertension is a major determinant of renal damage, affecting the delicate structure of of the kidneys^23^ and activating mediators that promote inflammation, fibrosis, and further glomerular injury^24^. As expected, diabetic patients with the selected disease duration exhibited significantly higher blood pressure levels compared to controls. To ensure accurate and controlled comparisons, all hypertensive diabetic patients were selected based on their use of the same antihypertensive medication, ACEIs. Furthermore, independent and specific comparisons were conducted among diabetic patients with and without albuminuria, as shown in Table (3). Patients were independently classified according to their hypertensive status to assess the impact of hypertension on diabetic cohorts with and without DN.

Regarding glycemic indices (FPG, A1C), a persistent state of significant hyperglycemia contributes to marked dyslipidemia, characterized by elevated levels of cholesterol and triglycerides alongside reduced HDL-c. The coexistence of chronic hyperglycemia and dyslipidemia promotes the generation of reactive oxygen species (ROS), leading to oxidative stress. This oxidative damage activates inflammatory pathways, stimulates immune cell activity, and enhances the release of pro-inflammatory cytokines. The resulting chronic inflammation further exacerbates kidney injury by promoting fibrosis and accelerating the loss of renal function. Together, oxidative stress and inflammation form a vicious cycle that significantly contributes to the progression of DN^25^. Additionally, persistent hyperglycemia has a direct detrimental effect on the glomerular microvasculature, ultimately leading to nephrosclerosis^26^.

As mentioned before, plasma high molecular weight KNG-1 is found to be increased with albuminuria as progressive renal function decline seen in diabetic patients^27^. Our results came also supporting with a significant increase in KNG-1 among patients with DN. The plasma kallikrein-kininogen system (KKS) plays a substantial role in regulation of coagulation and inflammation. Kallikrein is a highly abundant plasma protein that is activated by contact with negatively charged surfaces. When active, kallikrein cleaves one of its substrates, high molecular weight kininogen, resulting in release of bradykinin (BK). BK exerts its actions promoting vasodilation, vascular permeability and edema by stimulating cognate cell surface receptors, B1R and B2R^28^. The association of the KKS in DN has been examined extensively. In human studies, *Jaffa et al.*, studied plasma samples from patients enrolled in the Diabetes Control and Complications Trial and observed that plasma pre-kallikrein levels were correlated with hypertension, albumin excretion rates, and the development of albuminuria^29^.

HSPG consists of one or more heparan sulfate chains attached to a core protein^30^. Heparan sulfate chains are linear polysaccharides consisting of 50–200 repeating disaccharide units. Their biosynthesis is complex^31^ exhibiting considerable structural diversity within and among tissues. Within the kidney glomerulus, several types of HSPG are found with distinct distributions: while some types are associated with podocyte cell surfaces, most types are components of basement membrane^32,33^.

Our results showed that there is a significant increase in HSPG in diabetic patients compared to healthy subjects and a significant increase in diabetic patients with- compared to those without albuminuria. As documented earlier^34^, patients with diabetes showed elevated levels of plasma heparanase. Involvement of either podocyte cell surface or basement membrane HSPG families leads to endothelial dysfunction and loss of podocytes influences the glomerular filtration properties and finally resulting in DN. Such changes in HSPG levels are also supported by another study which also showed increased levels of heparanase in human kidney^35^. All these data suggest that heparanase may play a role in modifying HSPG structures in glomeruli, which might contribute to the development of DN.

Robo4 is a transmembrane receptor that belongs to the Roundabout (Robo) family of axon guidance molecules. Robo4 is an endothelial-specific receptor that participates in endothelial cell migration, proliferation, and angiogenesis and the maintenance of vasculature homeostasis^36^. Our results showed that there is a significant increase in Robo4 in type 2 diabetic patients cohorts compared to healthy subjects and a significant increase in type 2 diabetic patients with albuminuria compared to those with normoalbuminuria. Abnormal levels suggest dysregulation of angiogenesis and vasculature homeostatic mechanisms affecting glomerular vascular integrity causing lesions in glomeruli and finally leading to kidney dysfunction in diabetic patients^37^.

Hypertension is a common comorbid disease of diabetes and is a major risk factor for the development of macrovascular and microvascular complications of diabetes^29^. Although advanced DN secondarily worsens hypertension, the rising blood pressure may also be an early contributor to vascular damage. Accumulating evidence supports a relation between activity of the KKS and the development of hypertension and renal impairment. Two forms of kallikrein exist: one in tissue and another in plasma. Tissue kallikrein, which is mainly expressed in glandular tissue, kidney, vasculature, and brain, preferentially acts on low molecular weight kininogen substrate to release lysyl-bradykinin. Plasma kallikrein preferentially acts on high molecular weight kininogen substrate to release BK^38^. BK, the principal effector of the plasma KKS, is generated both systemically and locally within the vessel wall, where it acts in a paracrine or autocrine fashion to influence vascular tone ultrastructure^29^. Angiotensin I-converting enzyme, a zinc metallopeptidase widely distributed on the surface of endothelial and epithelial cells, plays a key role in the renin–angiotensin system and in the KKS. It catalyzes not only the conversion of angiotensin I to the vasoconstricting peptide angiotensin II, but also the conversion of active BK, kallidin, and kallidin-like peptide to inactive forms. So, the use of ACEIs significantly increases plasma levels of BK at doses much lower than those required to reduce angiotensin II levels^39^. Our study showed that all the diabetic hypertensive patients in the study took the same antihypertensive drug, which is ACEI, in which they have a role on the KKS. This may explains our results which showed significant increase in KNG-1 in hypertensive diabetic patients with albuminuria compared to normotensive diabetic patients with albuminuria as ACEI increase the plasma level of BK which is the precursors of KNG-1^40,41^. This is also in accordance with the literature as KNG-1 is converted into proinflammatory peptides known as kinin (BK and kallidin) via tissue or serum kallikrein^42^. Generally, the KKS plays a role in inflammation, prevention of apoptosis and oxidative stress^42^. Also, there is a significant increase in HSPG in hypertensive diabetic patients with albuminuria compared to normotensive diabetic patients with albuminuria due to another common feature of hypertension is vascular dysfunction^43,44^ which is intimately associated with impaired nitric oxide (NO) signaling. HSPGs are known regulators of vascular function. So, the effects of HSPG on the vasculature include the activation of NO signaling^45,46^. So, the HSPG has been shown to activate endothelial nitric oxide synthase (*eNOS*) in conditions of increased shear stress^47,48^. There is an increase in Robo4 in hypertensive diabetic patients with albuminuria compared to normotensive diabetic patients with albuminuria due to hypertension and diabetes affect the endothelial function causing endothelial dysfunction. Since hypertension causes vascular dysfunction, Robo4 is specifically expressed in endothelial cells. Robo4 mediates endothelial cell migration, proliferation, angiogenesis, and vascular stabilizations, so diabetes and hypertension affect Robo4 levels^42,49^. Because Robo4 is abundantly expressed in areas of active angiogenesis and maintains the vascular integrity, it may function as a biomarker or target for treatments for tumors, inflammation-related vasculopathy and diabetes-induced neovascularization^36^.

Upon performing correlation analysis among investigated parameters, results interestingly revealed that all investigated parameters were correlated significantly with both SBP and DBP, most notably in diabetic cases. This supports the hypothesis that hypertension could act as a common contributing factor enhancing the expression of all these proteins^40,41,43,44,49^.The interplay between vascular stress, endothelial dysfunction and hypertensive vascular injury could be an underlying mechanistic pathophysiology reflecting progressive endothelial activation and vascular changes seen in diabetic cases^50,51^. In the same manner, the key biomarker of renal glomerular permeability, UACR, is found to be strongly correlated with all investigated biomarkers especially in diabetic cohort reflecting the role of all these biomarkers in glomerular injury and altered basement membrane integrity seen in DN^29,34,35,37^.

Notably, results showed also significant correlations in-between investigated parameters. These strong interrelationships suggest pointing our minds toward shared mechanistic pathology involving vascular and endothelial damage. In contrast, results showed weak or insignificant correlations with A1C, a key biomarker of glycemic status, implying their involvement in vascular and glomerular dysregulation rather than in hyperglycemia. Collectively, all these data suggest that these biomarkers could be potential candidates for monitoring vascular injury and DN progression beyond glycemic mirrors.

Results of the study suggested a collaborative combined influence of both hypertension and albuminuria on the profile patterns of KNG-1, HSPG and Robo4 in diabetic patients. Therefore, we addressed 2 new aims; firstly, to study how the presence of hypertension, as contributing factor, could *independently* influence the susceptibility of DN progression “persistent albuminuria” as a complication in T2DM. Secondly, to study how the combined effect of both Hypertension and Albuminuria *together* could impact the expression profile pattern of the investigated biomarkers. In other words, we would like to understand the interaction between both Hypertension and Albuminuria in modulating the profile data of KNG-1, HSPG and Robo4.

For the first aim, results of 2 independent analyses proved the high impact of hypertension as an independent factor for developing DN among diabetic patients even if gender &/or glycemic status was kept constant or adjusted Padj. = 0.000.

Hypertension and albuminuria are both independent risk factors for cardiovascular and renal diseases. However, the combined effect of these conditions on biomarkers associated with endothelial dysfunction, renal damage, and vascular health has not been fully elucidated. This study aimed to investigate the combined impact of hypertension and albuminuria on three biomarkers KNG-1, HSPG and Robo4 using a two-way ANOVA approach. This analysis helps us understand whether the combination of these two factors leads to compounded effects on these biomarkers and their potential clinical relevance. Results revealed a significant interaction between hypertension and DN in modulating the levels of all three biomarkers investigated (KNG-1, HSPG and ROBO4). Results also confirm that these 2 factors do not act independently; rather, they have a combined, amplifying influence on biomarker expression, reflecting more severe endothelial and renal affection. Therefore, we can conclude that hypertension and DN seem to behave as vascular stressors in synergism. Now, if we go back through the results of our correlation analysis, we can find that results reinforce this conclusion. In correlation results, all the 3 biomarkers showed significant direct correlations with both SBP and DBP, especially within the diabetic cohort, indicating that blood pressure elevation plays a direct role in upregulating these markers. Similarly, strong correlations seen with UACR, a marker of DN, suggest that glomerular injury itself is tightly linked with increased biomarker levels. Thus, by integrating results of both analyses, the observed interaction between hypertension and DN in upregulating levels of KNG-1, HSPG and ROBO4, demonstrated through two-way ANOVA analysis, is further reinforced by the significant correlations of these biomarkers with SBP, DBP, as well as UACR. Clinically, this supports the concept that the coexistence of hypertension and DN exerts a synergistic effect on microvascular damage, as reflected in the increased biomarker profiles. These findings reinforce the role of these markers as potential integrative indicators of renal and endothelial stress, particularly in high-risk diabetic patients.

All the above results of the biomarkers and the effect of hypertension and albuminuria are in accordance with the literature that stated that hypertension promotes shearing stress and oxidative stress in endothelial cells. These injured endothelial cells lose the glycocalyx that covers their fenestrae; therefore, they cannot prevent albumin from moving through the basal membrane^51^. Accordingly, albuminuria is attributed to endothelial cell injury and affected podocytes. The juxtamedullary nephrons often face conditions characterized by intense hypertension due to vessel strain. Albuminuria, a well-known consequence of diabetes mellitus^52^ has served as an indicator of pressure-associated injury to nephrons^53^. Several disease conditions, as well as hypertension, also cause proteinuria in a similar manner^54,55^. The endothelial surface layer is diminished by the increased expression of heparanase, which is induced by oxidative stress^56^. In the setting of diabetes mellitus, this phenomenon may explain the albuminuria observed in DN. The negatively charged sugar chains attached to proteo-glycans mediate the permselective properties of glomerular endothelial cells^51^. High concentrations of glucose increase the expression of both dysfunctional endothelial nitric oxide synthase and ROS, whereas high doses of free fatty acids do not increase oxidative stress^50^, also there are several possible hypotheses that may explain the causality of hypertension as it relates to albuminuria. First, endothelial injury induces albuminuria in the kidneys and hypertension in the entire body. Such endothelial injury may be caused by cigarette smoking, diabetes or dyslipidemia, or by hypertension itself. This theory is similar to the theory depicting albuminuria as the egg to hypertension’s chicken. Based on this hypothesis, endothelial injury may be the grandmother of hypertension. Second, albuminuria occurring at the level of the glomeruli is mitigated via the resorption of albumin by the renal tubules^50^. The reabsorbed albumin induces oxidative stress in the renal tubules, resulting in kidney injury, a widely accepted cause of hypertension. The findings from this study underscore the importance of studying the interaction between hypertension and albuminuria in relation to biomarker levels. The results suggest that these two conditions, when present together, amplify the effects on biomarkers associated with endothelial dysfunction, renal damage, and vascular injury. The significant interactions observed for all three biomarkers (KNG-1, HSPG and Robo4) emphasizing the need for integrated clinical management strategies that target both hypertension and albuminuria simultaneously, particularly in patients at risk for cardiovascular and renal complications.

All these findings suggest that patients presenting with both hypertension and albuminuria may experience accelerated or amplified pathophysiological changes, emphasizing the importance of integrated management strategies targeting both risk factors simultaneously to potentially mitigate disease progression. Hypertension and albuminuria are both independent risk factors for cardiovascular and renal diseases.

Finally, results of ROC analysis revealed that KNG-1 seems not to be elevated due to diabetes itself, but it is highly responsive to persistent albuminuria as a strong indicator of kidney damage, making it a potential diagnostic biomarker for DN progression, not just diabetes. The same applies to Robo4 in that it has no significant utility in identifying diabetic status. But according to data obtained by ROC curves, Robo4 demonstrated good diagnostic performance in identifying albuminuria reflecting vascular endothelial injury that occurs secondary to DN, not diabetes itself. In contrast, ROC data for HSPG implies fair diagnostic utility in identifying diabetes itself suggesting some relevance to general diabetic pathology^57^. This likely indicates its ability to reflect early diabetic renal structural changes, even before clear albuminuria, but it performs best in established DN. Collectively, ROC analysis confirmed that while the studied biomarkers (KNG-1, Robo4) failed to differentiate diabetic patients from controls, they demonstrated strong discriminative capability in reflecting diabetic renal complications reinforcing what we discussed earlier about their role as indicators of renal microvascular damage among diabetic patients. These findings highlight their potential role in early diagnosis and/or staging of DN progression.

A key objective of the present study was to determine whether the investigated biomarkers provide additional clinical value beyond albuminuria, particularly in the context of early renal alterations. To address this, we performed supplementary analyses focusing on both normoalbuminuric patients and multivariable predictive modeling. Within normoalbuminuric subjects, Robo4 demonstrated a modest but significant association with GFR and emerged as a borderline independent predictor after adjustment for conventional risk factors. Although the magnitude of this effect was limited, these findings suggest that alterations in vascular-related biomarkers may precede overt albuminuria, reflecting early endothelial and microvascular dysfunction.

More importantly, multivariable logistic regression and ROC analyses demonstrated a substantial improvement in predictive performance when biomarkers were incorporated into clinical models. The marked increase in explained variance and the significant enhancement in AUC indicate that KNG-1, HSPG, and Robo4 provide complementary information beyond traditional clinical variables. These results support the concept that these biomarkers are not merely reflections of albuminuria status but rather integrative indicators of underlying vascular and renal pathophysiology.

Taken together, these findings suggest that the clinical utility of these biomarkers lies in their potential to enhance risk stratification when used alongside established markers, rather than replacing albuminuria. This is particularly relevant in early or subclinical stages of DN, where conventional markers may not fully capture ongoing pathological changes.

Future longitudinal studies are warranted to validate the predictive value of these biomarkers for renal function decline over time.

### Study limitations

This study has several limitations that should be considered. First, its cross-sectional design limits the ability to establish causal relationships or confirm the predictive value of the investigated biomarkers for future renal function decline. Second, DN was defined based on albuminuria status, which may constrain the assessment of whether these biomarkers provide independent diagnostic value beyond UACR, although additional multivariable analyses were performed to address this aspect. Third, subgroup analyses within normoalbuminuric patients were limited by relatively small sample size, particularly after further stratification, which may affect statistical power and the stability of the observed associations. Additionally, GFR was used as a surrogate marker for renal function decline and may not fully capture subtle early pathological changes. Another limitation relates to the use of albuminuria as the primary criterion for defining DN. Although widely accepted in clinical practice, albuminuria is not entirely specific for DN and may be influenced by other conditions such as hypertension, transient hemodynamic changes, or non-diabetic renal disease. Consequently, reliance on UACR alone may introduce some degree of misclassification, potentially affecting the interpretation of biomarker performance. This further underscores the need for complementary biomarkers that reflect underlying renal and vascular pathology beyond albuminuria. The use of ACE inhibitor therapy among hypertensive patients may also have influenced biomarker levels especially KNG-1, particularly those related to the kallikrein–kinin system. Finally, the single-center design may limit the generalizability of the findings.

Despite these limitations, the study provides important insights into the potential complementary role of KNG-1, HSPG, and Robo4 in improving risk stratification of DN beyond traditional clinical parameters.

## Conclusion

This study demonstrates the significant and synergistic impact of the concurrent presence of both hypertension and albuminuria on the expression profile patterns of three critical biomarkers, KNG-1, HSPG and ROBO4. Clinically, these findings suggest that both conditions should be carefully managed together in diabetic patients to mitigate the compounded risks associated with elevated biomarker levels, which could indicate early signs of cardiovascular and renal dysfunction. The interaction between hypertension and albuminuria significantly alters biomarker levels, particularly for markers of endothelial and vascular damage, emphasizing the need for comprehensive management of both conditions in clinical practice. Further research into these biomarkers and their combined effects could help refine risk stratification and improve treatment strategies in individuals with concurrent hypertension and albuminuria. Finally, it is very recommended that strict control of hypertension is mandatory to prevent or at least delay progression of DN and cardiovascular complications in T2DM.

This study demonstrates that the serum biomarkers KNG-1, HSPG, and Robo4 are significantly associated with renal involvement in patients with T2DM and are influenced by the combined presence of hypertension and albuminuria. Beyond their association with established markers, the present findings highlight that these biomarkers provide substantial incremental value when integrated with conventional clinical parameters, as evidenced by the marked improvement in multivariable model performance and discriminative accuracy.

Importantly, additional analyses within normoalbuminuric subjects suggest that alterations in these biomarkers—particularly Robo4—may reflect early vascular and renal functional changes prior to overt albuminuria, although this effect appears modest and warrants further investigation. These findings support the concept that KNG-1, HSPG, and Robo4 act as integrative indicators of underlying endothelial and microvascular dysfunction rather than merely mirroring albuminuria status.

Collectively, the results indicate that these biomarkers may serve a complementary role in enhancing risk stratification and identifying patients at higher risk of early renal alterations when used alongside established clinical markers, rather than replacing them. This integrative approach may be particularly valuable in capturing subclinical disease processes not fully reflected by albuminuria alone.

Future longitudinal and multi-center studies are required to validate the predictive utility of these biomarkers for renal function decline and to determine their potential role in clinical decision-making and early intervention strategies.

## Data Availability

The data sets used and/or analyzed during the current study are available from the corresponding author on reasonable request.
